# piRNN: deep learning algorithm for piRNA prediction

**DOI:** 10.7717/peerj.5429

**Published:** 2018-08-03

**Authors:** Kai Wang, Joshua Hoeksema, Chun Liang

**Affiliations:** 1 Department of Biology, Miami University, Oxford, OH, USA; 2 Department of Computer Science & Software Engineering, Miami University, Oxford, OH, USA

**Keywords:** piRNA, Deep learning, Convolution neural network

## Abstract

Piwi-interacting RNAs (piRNAs) are the largest class of small non-coding RNAs discovered in germ cells. Identifying piRNAs from small RNA data is a challenging task due to the lack of conserved sequences and structural features of piRNAs. Many programs have been developed to identify piRNA from small RNA data. However, these programs have limitations. They either rely on extracting complicated features, or only demonstrate strong performance on transposon related piRNAs. Here we proposed a new program called piRNN for piRNA identification. For our software, we applied a convolutional neural network classifier that was trained on the datasets from four different species (*Caenorhabditis elegans*, *Drosophila melanogaster*, rat and human). A matrix of *k-mer* frequency values was used to represent each sequence. piRNN has great usability and shows better performance in comparison with other programs. It is freely available at https://github.com/bioinfolabmu/piRNN.

## Introduction

Piwi-interacting RNAs (piRNAs) are the largest class of small non-coding RNAs that are enriched in animal germline cells (Lau et al., 2006). piRNAs were thought to be transposon silencers in germline cells to maintain genome integrity through epigenetic and post-transcriptional regulations (Aravin, Hannon & Brennecke, 2007). Recent studies reveal that piRNAs are found in somatic cells and are also related to certain mRNAs degradation (Malone et al., 2009; Rouget et al., 2010). This suggests that piRNAs may have more important functions in germline and somatic cells that are waiting for further exploration. The first step in studying the function of piRNAs is to identify them from other small non-coding RNAs like miRNA, siRNA, etc. Experimental approaches like extracting piRNAs or obtaining transposon-related small RNAs from germline cells have been applied to identify piRNAs (Aravin et al., 2007; Lau et al., 2006). Since piRNAs are not only expressed in germline cells and their functions are not limited to silencing transposons, those experimental approaches have limitations (e.g., incapability of capturing piRNAs with low expression levels and exploring somatic piRNAs). Therefore, computational approaches were proposed to predict piRNAs from small RNA sequence data (Betel et al., 2007).

The first bioinformatics tool for piRNA prediction was created by applying Fisher discriminant algorithm to *k-mer* sequence features using small RNA data (Zhang, Wang & Kang, 2011). This algorithm can achieve a precision of >90% and a sensitivity of >60% in five species data (nematode, fruit fly, human, rat, and mouse). Piano (Wang et al., 2014) and piRNAPredictor (Li et al., 2016) were developed to predict transposon-related piRNAs by using support vector machine and weighted ensemble method, and they achieved a very high accuracy of over 95%. Another transposon-related piRNA prediction program that developed using ensemble learning method also achieved great performance (Luo et al., 2016). A previous study (Beyret, Liu & Lin, 2012) suggested that only a portion of piRNAs are related to transposons, so the aforementioned three programs are only suitable for a subset of total piRNAs whereas a large portion of piRNAs would be ignored by these programs. In addition, other programs were also developed to predict total piRNAs from small RNA data. piRPred (Brayet et al., 2014) used a support vector machine and kernel combination strategy to achieve over 86% accuracy on human data and over 89% accuracy on fruit fly data. In piRPred, sequence information (i.e., *k-mer* and 5′ uridine) and genome information (i.e., distance to pericentromeric and subtelomeric regions and piRNA cluster location) were extracted and combined together as sequence features for SVM training and testing. 2L-piRNA (Liu, Yang & Chou, 2017) is a two-layer ensemble classifier for piRNA prediction and functional annotation, with the accuracy of 86.1%. Since the accuracy/specificity of piRPred and 2L-piRNA is around 85%, there is still room for improvement in piRNA prediction. Pibomd (Liu, Ding & Gong, 2014) is also a piRNA prediction program established on a support vector machine by using sequence motif features. The sensitivity and specificity of Pibomd are around 90%. Since Pibomd is trained only on mouse data, it cannot be used for other species. IpiRId (Boucheham et al., 2017) combined sequence content features and epigenomic features in piRNA prediction and achieved around 90% accuracy on human, mouse, and fruit fly data. However, its feature extraction is complicated and the epigenomic data in addition to small RNA data is also needed, making IpiRId difficult to apply on non-model organisms. V-ELMpiRNAPred (Pian et al., 2017) was developed for human piRNA prediction with a high specificity(95%) and sensitivity (94%). Unfortunately, it is difficult to apply V-ELMpiRNAPred to other model organisms.

Recently, deep learning is a very popular and widely used technique for many kinds of classification tasks (LeCun, Bengio & Hinton, 2015). Deep learning algorithms have been utilized for several bioinformatics applications. For example, DeepBind was developed for predicting DNA- and RNA-binding proteins (Alipanahi et al., 2015) and DeepChrome for predicting gene expression levels from histone modification data (Singh et al., 2016). In this paper, we propose a new program called piRNN for piRNA prediction that was developed by using a deep learning model based on convolution neural network (CNN) framework. Our program achieved over 90% accuracy on *Caenorhabditis elegans*, *Drosophila melanogaster*, rat and human data.

In order to develop a powerful piRNA prediction program for biologists to find piRNA accurately from small RNA data, there are five critical questions need to be addressed properly as follows (Chou, 2011): How to select valid datasets to train and test the predictor; how to use effective mathematical expression to represent biological sequences; how to develop a powerful algorithm to operate the prediction; how to perform cross-validation tests properly to evaluate the predictor; and how to make the predictor user-friendly and can be easily accessed by the public? In piRNN, the above five questions are adequately addressed and described in details in the following parts.

## Materials and Methods

### Data sets

In our program, *C. elegans*, *D. melanogaster*, rat and human piRNAs were used for training models. *C. elegans*, rat and human data were downloaded from piRBase (Zhang et al., 2014). *D. melanogaster* data were downloaded from piRNABank (Lakshmi & Agrawal, 2008). In total, 28,219 *C. elegans* piRNA sequences, 22,336 *D. melanogaster* piRNA sequences, 63,182 rat piRNA sequences, and 32,826 human piRNA sequences were used as the positive datasets. To develop a powerful DNA/RNA sequence classifier, one of the most critical and challenging problems is how to generate a valid negative dataset that can be used effectively in model training and testing. Previous researches such as PseKNC (Chen et al., 2014) and Pse-in-One (Liu et al., 2015) provided comprehensive methods that can be used to generate pseudo DNA/RNA sequences (i.e., negative dataset) for computational biology research. Based on the previous study (Brayet et al., 2014), the negative datasets of each species consist of three parts: (1) corresponding fake-piRNA sequences generated using the first order Markov model where the probability distribution came from the positive datasets, (2) corresponding mature microRNA sequences downloaded from miRBase (Kozomara & Griffiths-Jones, 2014), and (3) tRNA fragments of certain lengths randomly cut from tRNA sequences downloaded from Genomic tRNA database (Chan & Lowe, 2009). Accordingly, both positive and negative datasets have the same sequence numbers, respectively, for each species. Each species’ dataset, both positive and negative data, was divided into two parts: training-testing dataset (90%) and validation dataset (10%). All models were trained using 10-fold cross validation with the training-testing data set. The validation dataset was used for comparison with other programs. More details can be found in the Supplemental File.

### Sequence feature extraction

**Figure 1:**
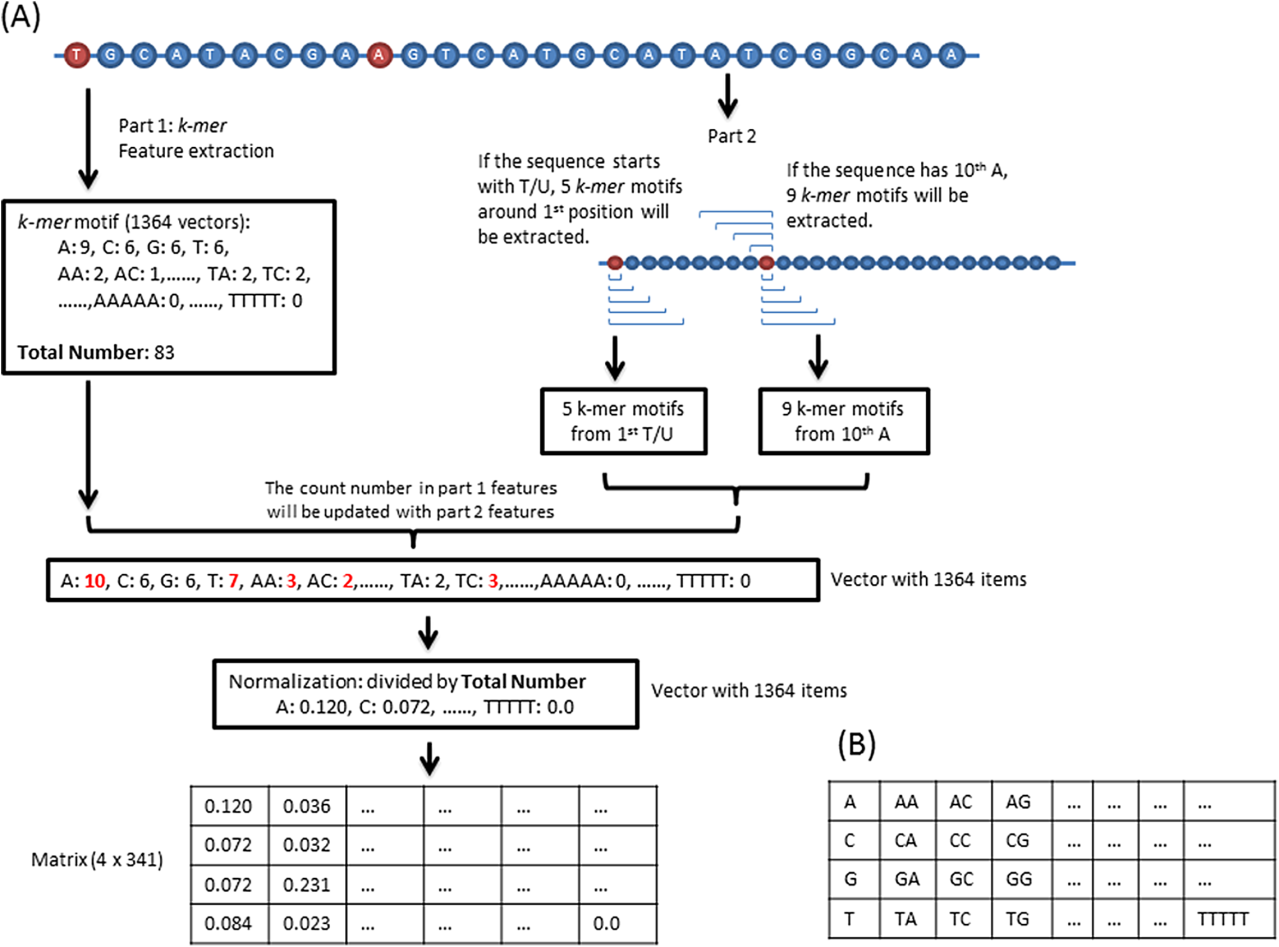
Feature extraction. (A) Sequence features consist of two parts. The first part is the *k-mer* motifs of the whole sequence. The second part *k-mer* motifs are around the first T/U and/or 10th A. If a sequence dose not start with a T/U or do not have a 10th A, the corresponding second part will not be calculated. (B) The 1,364 vectors are transformed into this matrix.

Previous studies demonstrate that sequence-derived features can be efficiently used for developing different types of DNA/RNA sequence classification programs (Zhang et al., 2017a, 2017b). In our program, the sequence feature consists of two parts. The first part is *k-mer* (*k* = 1, 2, 3, 4, 5) motif frequencies. In total, 1,364 (sum of 4^1^, 4^2^, 4^3^, 4^4^, and 4^5^) values were extracted from each sequence. For the second part, we counted the *k-mer* motifs around the first and 10th base because piRNA has a high probability starting with a “T/U” and containing an “A” at 10th position (Hirakata & Siomi, 2016). Specifically, if the sequence starts with a “T/U” and/or has an “A” in the 10th position, then we updated the second part feature vectors into the first vectors as shown in Fig. 1. For training, the total 1,364 vectors were transformed into a 4 × 341 matrix (Fig. 1).

### CNN architecture

**Figure 2:**
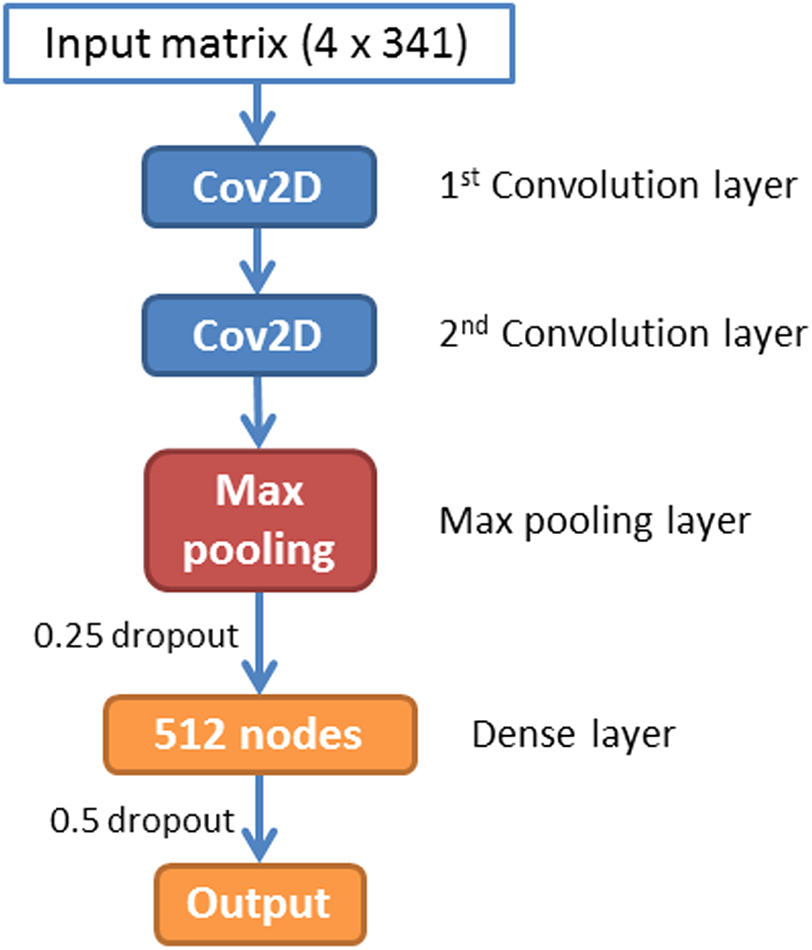
Architecture of the convolution neural network.

A CNN is a feed-forward artificial neural network that has been widely used in image recognition. In this paper, we have implemented a CNN for piRNA identification by using TensorFlow (Abadi et al., 2016) and Keras (Chollet, 2015). The whole program was implemented in Python 3.5. The architecture of piRNN is showed in Fig. 2. The input of each sequence is a 4 × 341 matrix. Each element of this matrix represents *k-mer* motifs frequencies as shown in Fig. 1. Two one-hot vectors ([0, 1], [1, 0]) were used to label the piRNA and non-piRNA sequences. The first part of our network is two convolution layers. A total of 32 2 × 2 filters were used in these two convolution layers to scan the input matrixes. The second part is a max pooling layer with 2 × 2 pooling size. A dropout layer with 0.25 dropout ratio is connected to the max pooling layer. The third part is a dense layer with 512 nodes. We applied a 50% dropout after the dense layer to prevent over fitting. The last layer is the outputs layer with two nodes that correspond to the piRNAs and non-piRNAs.

### Model training

A total of 10-fold cross validation was used for model training in four species individually. A small batch size of 32 was applied in all training processes. During the 10-fold training for all four species, the training process will be stopped if the value of cost function did not decrease after 10 epochs. Since the input of our program is a small matrix (4 × 341) and it is easy to get overfitting in CNN training, we used a simple CNN architecture that only contains two convolution layers, one max pooling layer, and one dense layer, with two dropout layers (see Fig. 2). All parameters used in training were set in terms of the default values from Keras document (https://keras.io/). The four species models were trained by using the same CNN architecture because we are aiming to establish a piRNA prediction tool that can be easily used by biologists. It is worth mentioning that piRNN allows re-training to get better models if more data is available.

## Results

### Classification performance

**Figure 3:**
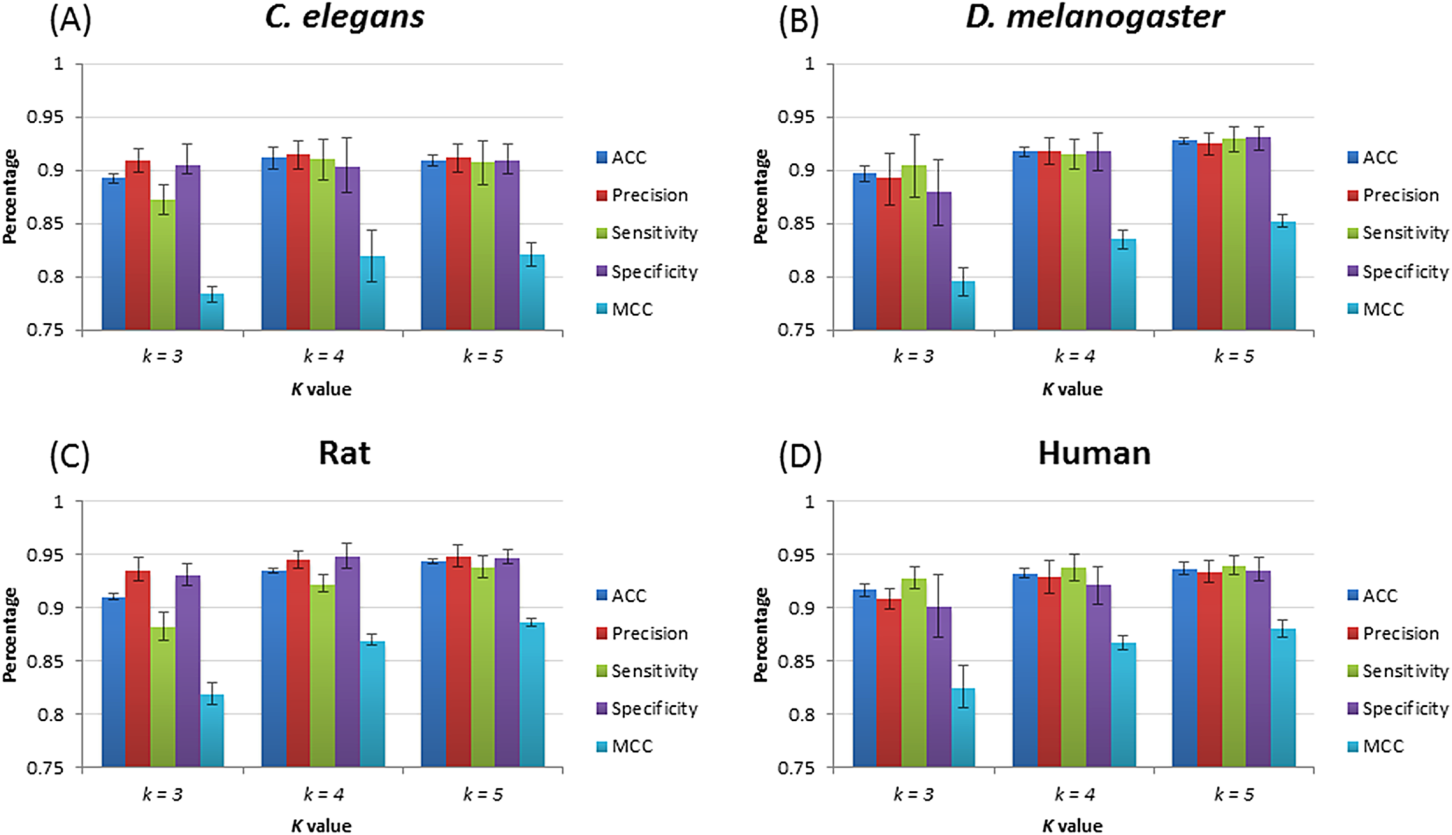
piRNN performance with different *k* value for different species. (A), (B), (C), and (D) show the results of *C. elegans*, *D. melanogaster*, rat, and human, respectively.

After training, four species–specific models are tested using their relevant test datasets to evaluate model performance. Figure 3 shows the performance of piRNN. Accuracy (ACC), precision (*Pre*), sensitivity (*Sn*), specificity (*Sp*), and Matthews correlation coefficient (MCC) were used for performance evaluation. These measurements were calculated from the testing datasets by 10-fold cross validation. Since piRNA is small non-coding RNA only with ∼30 nt in length, a large number of *k* in feature extraction would produce many useless features (value equals 0). Based on the testing results and taking into account the calculation cost and operation time, we selected *k* = 5 lastly for our program.

### Comparison with other methods

Previous piRNA prediction/identification programs can be classified into two categories or groups. The first one is those that only focused on predicting transposon-related piRNAs, such as piRNAPredictor (Li et al., 2016), Piano (Wang et al., 2014), etc. These programs usually can achieve very high accuracy and precision. Since the transposon-related piRNAs are only a small portion of total piRNAs, especially in mammals, it is easy to train a model with good performance on small datasets. In this context, non-transposon-related piRNAs (over half of total piRNAs) would be excluded from the prediction results (Beyret, Liu & Lin, 2012). Since piRNAs are also showed to silence certain mRNAs (Rouget et al., 2010), these programs have obvious limitation in piRNA prediction. The second group of programs can be used to predict total piRNAs from small RNA data. Example include piRNApredictor (Zhang, Wang & Kang, 2011), piRPred (Brayet et al., 2014), and IpiRId (Boucheham et al., 2017). Combined with genomic features like piRNA genome locations and epigenetic data, piRPred and IpiRId achieved better performance than the piRNApredictor in piRNA identification. Since they need genomic features for prediction, it is hard to apply these programs to non-model organisms that lack enough genomics resources.

For performance comparison, we compared our program with Piano (Wang et al., 2014), 2L-piRNA (Liu, Yang & Chou, 2017), and piRNApredictor (Zhang, Wang & Kang, 2011). Piano is the first piRNA prediction program that only focused on transposon-related piRNAs. piRNApredictor is the first software that can predict piRNAs using small RNA-Seq data. piRPred (Brayet et al., 2014), Pibomd (Liu, Ding & Gong, 2014), V-ELMpiRNAPred (Pian et al., 2017), and IpiRId (Boucheham et al., 2017) are web-based tools that are unable to execute locally. piRNAPredictor (Li et al., 2016) does not provide any standalone downloadable programs. Therefore, we only selected Piano, 2L-piRNA, and piRNApredictor for the performance comparison. A pervious study shows that *D. melanogaster* has different length distribution from mammal piRNAs (Wang et al., 2014), so we only used human data that is representative of mammal piRNAs and *D. melanogaster* piRNAs for comparison. Our comparison results are listed in Table 1. piRNN shows the best performance for all five measurements for both species, with accuracy, precision, sensitivity, specificity, and Matthews correlation coefficient of over 90% (see Table 1). Compared to the results provided in the paper of IpiRId (accuracy for human and fruit fly is 90.09 ± 0.25 and 92.59 ± 1.87, respectively), our program piRNN also demonstrated better performance results than IpiRId on both fruit fly and human data. Considering the fact that our program is the one with the best performance and only utilizes *k-mer* features without other data (e.g., genome location or epigenetic data) in piRNA prediction, piRNN is more user-friendly for biologists to apply on new data. Clearly, piRNN is a useful tool for piRNA prediction in non-model organisms where genomics resources are limited.

**Table 1:** Comparison between Piano, piRNApredictor, 2L-piRNA, and piRNN. The results of piRNN are highlighted in bold.

Program	Species	ACC	*Pre*	*Sn*	*Sp*	MCC
Piano	Fruit fly	0.68 ± 0.021	0.63 ± 0.018	0.87 ± 0.021	0.50 ± 0.034	0.40 ± 0.043
	Human	0.62 ± 0.013	0.58 ± 0.015	0.92 ± 0.008	0.32 ± 0.016	0.30 ± 0.02
piRNApredictor	Fruit fly	0.53 ± 0.013	0.66 ± 0.066	0.14 ± 0.025	0.93 ± 0.012	0.11 ± 0.043
	Human	0.72 ± 0.019	0.84 ± 0.019	0.55 ± 0.04	0.89 ± 0.014	0.47 ± 0.032
2L-piRNA	Fruit fly	0.52 ± 0.027	0.65 ± 0.039	0.39 ± 0.035	0.71 ± 0.30	0.10 ± 0.051
	Human	0.67 ± 0.028	0.68 ± 0.031	0.79 ± 0.025	0.51 ± 0.042	0.31 ± 0.055
piRNN	Fruit fly	**0.95 ± 0.003**	**0.93 ± 0.006**	**0.97 ± 0.004**	**0.97 ± 0.004**	**0.90 ± 0.006**
	Human	**0.95 ± 0.004**	**0.94 ± 0.008**	**0.97 ± 0.012**	**0.97 ± 0.012**	**0.91 ± 0.008**

**Note:**

ACC, Accuracy; *Pre*, precision; *Sn*, sensitivity; *Sp*, specificity; MCC, Matthews correlation coefficient.

## Conclusions

In summary, we developed a deep learning program for identifying piRNAs based on CNN. The major advantages of this program can be concluded as: (1) this is the first deep learning based piRNA identification program that demonstrates the best performance in comparison to three previous programs, (2) this program adopts a genome-independent approach that does not need genome and/or epigenomic data for identifying piRNAs, (3) our program is an easy-to-use program with an option to choose one of the four well-trained models for four different species (*C. elegans*, *D. melanogaster*, rat and human), and (4) we also provide the training protocol and procedure for users if they want to retrain existing models or train new models for new species and conduct predictions. Our program is freely available at https://github.com/bioinfolabmu/piRNN. User can download all source codes and testing datasets from the aforementioned website. As pointed out in recent publications (Chou & Shen, 2009; Lin et al., 2014; Song et al., 2018), user-friendly web-service represents the future direction for computational tools. In future, we would like to establish a web-service for piRNN so that it is within the reach of general biologists. All updates of our program will be released in our github website. We are committed to improve piRNN for better piRNA prediction for more species in the future.

## Supplemental Information

10.7717/peerj.5429/supp-1 Supplemental Information 1 Data for training and testing.

## Additional Information and Declarations

### Data Availability

The following information was supplied regarding data availability: GitHub: https://github.com/bioinfolabmu/piRNN.
